# Toxicovigilance Systems and Practices in Africa

**DOI:** 10.3390/toxics4030013

**Published:** 2016-07-28

**Authors:** Pouokam Guy Bertrand, Hatem Abdel Moniem Ahmed, Randolph Ngwafor, Chiara Frazzoli

**Affiliations:** 1 Food Safety Laboratory, Biotechnology Center, University of Yaounde 1, Cameroon; 2 Department of forensic chemistry, Naif Arab University for security sciences, Riyadh 11452, Saudi Arabia; 3 Laboratory for Public Health Research Biotechnologies, Biotechnology Center, University of Yaounde 1, Cameroon; rangwafor@yahoo.com; 4 External Relations Office, Istituto Superiore di Sanità, Rome, Italy; chiara.frazzoli@iss.it; 5 Nutrition, Food Safety and Wholesomeness. Prevention, Education and Research Network (NOODLES), Via Mancinelli, 100, 00199 Rome, Italy

**Keywords:** toxicovigilance, toxicosurveillance, poisoning, Africa consumer, risk management

## Abstract

African consumers and citizens are growingly aware of the wide range of toxic poisoning scenarios from different products and hazards. Recurrent episodes on poisoning that have been reported in Africa include toxic hazards in consumers’ products ranging from food to herbal medicine, drugs, and cosmetics. Chemical poisoning remains an issue that is overlooked by public health stakeholders in Africa. Available information on toxicovigilance systems and practices in African countries is reviewed in terms of increasing development, organization and articulation levels. Less than nine out of 54 African countries have a legally recognized toxicovigilance system. Of these, the majority have created toxicovigilance systems recently, and are facing many challenges in developing them, at regional and country levels. Basic structures for a good toxicovigilance system include a phone line service (available 24/7), and hospital facilities. Pesticides emerge as the hazard recognized by all of the toxicovigilance systems, and may represent a prototypic toxicant towards a toxicovigilance system that is inclusive of a wider spectrum of toxicological hazards for the protection of community health. Toxicovigilance today is more reactive than preventive in Africa, but some milestones are present that constitute some promising seminal efforts.

## 1. Introduction

African consumers and citizens are growingly aware of the wide range of toxic hazards posed by living scenarios and consumers’ products. Toxic poisoning is defined by the World Health Organization (WHO) as all incidents caused by toxic substances originating from outside or inside the body [1]. According to WHO, Toxicovigilance (TV) is the active process of identifying and evaluating the toxic risks existing in a community, and evaluating the measures taken to reduce or eliminate them. Its role is to determine whether poisoning is caused by specific agents or circumstances, or whether specific populations suffer from a high incidence of poisoning. Toxicovigilance’s main goal is therefore to feed risk management with updated information for reducing the incidence of poisoning in a community by identifying, assessing and effectively/efficiently managing new, re-emerging, and known toxicological risks.

A comparison of events already reported across African countries (Table 1) revealed different poisoning scenarios. Some major causes include: accidental poisoning from mishandling of pesticides at home or in the farm [2,3], from kerosene ingestion due to unsafe storage at home [4], intentional ingestion of herbal potions of unknown composition [5], overdose of pharmaceuticals [6], drug suicide [7], snake bites [8], unsafe alcohol consumption, and accidental food poisoning [9]. Pesticides in foods of vegetal origin, unknown hazards in herbal medicines, petroleum in household products and toxics in food from restaurants are top on the list of poisoning past history. Toxicovigilance systems in African countries are still nascent but their role is now largely recognized. 

Based on recurrent poisoning scenarios reported in Africa, this paper describes the structure and organization of existing national TV systems in order to analyze their evolution towards the WHO recommended TV model. Toxicovigilance practices in countries without a formal TV system are also considered. 

## 2. National Toxicovigilance Systems in Africa

Basically, a TV system should be able to early identify, collect, process, evaluate, communicate and alert authorities and the public for preventive actions. A TV system is created and organized through a legal regulating document (Ministerial decree or Decision) that engages specific tasks for the implementation of a surveillance plan for chemical poisoning. The regulating document also defines communication aspects, i.e., how to manage information. This legal frame identifies a dedicated poison control center (PCC), or delegates an existing structure.

In its organization, a TV system comprises administrative and technical bodies. The technical body collects information on reported poisoning or suspected cases, conducts periodic risk assessment of chemicals, carries out research and epidemiology during outbreaks, and furnishes scientific advice to fuel the decision making process. 

In the perspective of global health governance (C Frazzoli, A Mantovani, R Esposito. Sustainable food safety and toxicant zoonoses: new prevention challenges in global health governance. Quaderni della Società Italiana di Medicina Tropicale e Salute Globale2016, 1: 117–127.), new tools may facilitate networking and harmonization of procedures. For instance, the WHO INTOX Data Management System is a sophisticated TV tool that enables fast analysis of the data collected by a PCC or the structure in charge of TV. The WHO INTOX Data Management System helps to find substances and products by commercial name chemical name, or chemical abstract registry (CAS) number. It can also be used to find the number of enquiries received about a specific substance in one or more age groups; to find the number of severely poisoned cases; and to find the number of cases who were given a specific treatment. The INTOX data management TV system has a well-defined set of standard reports designed to meet the broad needs of poisons information centers (P.I.C). Moreover, harmonized reports have been designed for the system, but it is also possible to customize reports to meet a center’s specific needs [1]. 

The following sections describe the different maturity levels of TV systems currently put in place in Africa: from spare toxico-surveillance practices (the case of Cameroon) and toxicology information centers (the case of Benin)**,** to poison information centers (the cases of Ghana, Kenya, South Africa and Zimbabwe) and poison control centers (the case of Algeria, Morocco and Senegal).

### 2.1. Toxico-Surveillance Practices: The Case of Cameroon

Cameroon is among that countries that have not yet established a formal TV system but already executes TV practices. For instance, the Ministry of Public Health has established an integrated disease surveillance and response system for more than 47 infectious diseases, which are subject to systematic notification. Some of these diseases include cholera, meningitis, measles, bird flu, yellow fever, malaria, arbovirus and rabies. Law No. 006 of 16 April 2001 provides the nomenclature and list of communicable livestock diseases subjected to obligatory notification [26], as well as of chronic diseases (diabetes, obesity, and hypertension). Weekly notifications are made using case report forms available at the three levels of the health pyramid (Central/strategic level, Intermediate/technical level, and Peripheral/operational level). In 2010, Cameroon had 178 health districts, with 154 functional district hospitals. Despite the good health data collecting system, it is difficult to produce poisoning case management and prevention information [27]. A second example of a functional surveillance program is the one set up for cholera in 2011. Giving the periodic occurrence of cholera outbreaks (2010–2012), cholera coordination control centers have been created. In this model, district hospitals nationwide are equipped with phone lines in order to communicate suspicious cases on a daily basis to a centralized call center located in the regional headquarters. Collected cases at regional level are analyzed by health personnel in charge of providing emergency response to victims and alert action at the national coordination office [9]. Unfortunately, this ongoing surveillance program does not cover poisoning cases caused by toxic chemicals, but can represent a prototype. Surveillance of drug intoxications and side effects are implemented through a pharmacovigilance system involving hospitals, the national laboratory of drugs and pharmaceutical product analysis, and the pharmacovigilance service based at the Ministry of Public Health. Functioning of this system is explained by the legal requirement that drug producers and distributors need to meet before the entry of a product in the national market. Post-marketing outcomes are monitored through pharmacovigilance actions such as monitoring of drug efficacy and occurrence of potential side effects for early interventions.

No formal TV system is yet established in Cameroon. In emergency care units of local hospitals, poisoning cases are managed and documented in hospital archives. There is no unique format to register new cases across hospitals. Neither is there an obligation for health professionals in hospitals to systematically notify new cases to a structure in charge of data collection, analysis and alert actions. 

Though there is no standard declaration format to record and systematically notify poisoning cases, hospital data in archives can be accessed, but only after a granted authorization. Toxicovigilance activities are most often carried out after an outbreak poisoning episode occurs. In such cases, surveillance is carried out; retrospective epidemiological investigations are done with available information in order to respond to the emergency. Toxicovigilance today is more reactive than preventive. Data on intoxication cases can be accessed in hospital and laboratories records, and case studies are published in medical and others scientific specialized journals. Police stations constitute another source of intoxication declarations, even if the quality of data retrieved may not be as details as the ones at PIC/PCC. Cameroon has a good number of structures involved in chemical surveillance, but there is no coordination between them. Data are collected from different inspections made by competent administrations from the Ministries of public health, trade, agriculture and fishing, as well as by the health and phytosanitary agents posted at the entry points of the country. Private industries, e.g., in the agro-food sector, can generate data from self-monitoring activities. 

No national database of toxic substances is constituted. No official report is published. If a hospital estimates that an outbreak or the situation looks alarming, then, it can decide to send and alert to the ministry of public health for information. 

Public control laboratories and university laboratories exist and analyze chemicals in food: noticeably, slaughtering houses gather data during ante and post mortem examination for quality control procedures of meat products; data collected (animal diseases, meat contamination and residues) are kept in a register and sent to the ministerial department in charge of slaughtering houses. However, these important raw data are not fully exploited for surveillance and public health purposes. 

Setting a coordinating mechanism or body could collect and process all potential surveillance data coming from the various structures and build a strong and functional national integrated surveillance mechanism at country level.

### 2.2. Toxicology Information Center: The Case of Benin 

The National Toxicology Information Center in Benin was created in early 2000 and established between 2000 and 2002 at the National University Hospital of Cotonou, after the identification of major toxic risks in different communities, prioritization of activities, and sensitization and involvement of stakeholders. The main function of the center is to provide expertise in toxicology as well as recommendations for poisoning management, TV, research, education and training in prevention and treatment of poisoning. Among major groups of poisoning chemicals covered by the Center are pesticides (organochloride and organophosphorus), insecticides, hydrocarbons in petroleum products, counterfeit drugs, and toxic medicinal plants. Beside consumers’ products, air pollution, drug abuse and allergy, and envenomation are also covered. Snakebites are frequent and have been found to be a leading cause of consultation in rural areas and a leading cause of reanimation in urban hospitals.

Emergency services available to the public are still to be developed, and even when some facilities exist at the level of the hospital for patient management, others risk factors, among which transport, play critical roles in preventing timely arrival of patients. In the northwest region, for instance, snakebites cause major threats to families. It is reported that 58% of patients reach the hospital less than 1 h after the incident, and 12% reach the hospital 3 h later [28]. Between 1995 and 1996, more than 120 villagers of the region died in the forest after being bitten by adder snakes; 600 other victims survived after hospitalization [29]. 

A free phone line number is reachable 24 h a day, seven days a week. People can call for inquiries and request for information on toxic substances and first emergency actions in case of intoxications. A call responder is available to register the inquiry and ensure follow up of each case.

Because of the recurrence of endosulfan poisoning in workers of the cotton producing region of Tchaorou, health authorities set up an additional specific outdoor surveillance system to ensure the reporting and management of all case pesticide poisoning. This outdoor system pivots on hospitals and health centers in the region, provides first aid treatment and alerts the reference regional hospital in case of suspicion of a pesticide poisoning. Cell phone and ordinary mail services are available to ease communication. Different scale grades are used in case classification: a case is “suspected” when an individual living in the Tchaorou health zone shows digestive and neurological pathologies after consumption of foodstuffs, while the case is said to be “probable” when the patient meets the criteria of a suspected case and investigation reveals important contact with pesticides. Health personnel and members of the community are educated to identify signs of pesticide poisoning and encourage reporting any suspected case to the nearest health center [30]. A major challenge of the system is the confirmation of probable cases. For the management of probable cases, collaboration with health centers and police services allows the free transport to the hospitals that are equipped with antidotes (atropine and conthration). The regional hospital has an emergency kit for deeper treatment; a special fund is donated to start treatment and reduce expenses for the patients. While taking care of suspected cases, field investigation and community sensitization campaigns are launched simultaneously. A standard collection form to register new cases is already designed, printed and available for the purpose. Moreover, a database of chemical substances present in commercial products is made available in a given region and kept at the documentation unit [27].

The National Public Health Laboratory (LNSP) has the obligation to declare all diseases listed among those subjected to obligatory notification. A crucial problem of the current legal framework is the weakness of the legal framework to declare collective food toxinfection (TIAC) cases: this hampered detailed investigations in case of potential outbreak. Some poisoning cases are declared in police stations [31]. 

Sensitization campaigns are made through mass media (radio and grassroots communities) in local languages. Antidotes and first aid drugs are provided to local hospitals for people to rapidly have access in case of need [32]. Exploitations of hospitals’ records are used to strengthen the decision making process and evaluate the efficacy of existing surveillance strategies through periodic consultation between all involved parties.

### 2.3. Poison Information Center: The Cases of Ghana, Kenya, South Africa and Zimbabwe 

The Ghana Poison Information and Consultation Centre, has been operational since 2002 and is under the umbrella of the Occupational and Environmental Health Unit of the Ghana Health services. Physically, the center is located both at the Ridge Hospital and at the regional hospital for the greater Accra Region. Before starting the poison information and consultation center, training support activities were proposed to future health personnel. For three consecutive years, health sector staff participated in workshops on the International Program for Chemical Safety INTOX data information system (IPCS’s INTOX). Familiarization visits were also organized at the Tygerberg (Cape Town, South Africa Republic) and Munich (Germany) PCCs. The call hours are 24 h a day, seven days a week. 

Major roles of the center include: (i) training of agricultural personnel in prevention and first aid management of pesticide poisoning; (ii) collection and analysis of poisoning data reported in health structures and TV activities; (iii) public awareness education and information programs; and (iv) development of training materials for health workers and for public education.

Between the main challenges of the center are the phone line difficulties, the lack of clinical laboratories to undertake toxicological analysis, and the weak TV system [5]. Multiple data collection sources are used, from hospital and laboratory records, to the declarations received at the call center. Research data retrieved from medical journals, institutional reports and other scientific peer review journals are exploited to understand the dynamic of poison-related diseases. The Ghana health services receive periodically a report on poisoning profiles nationwide in order to support the evaluations of objectives, identification of corrective actions and policy. 

The Agrochemicals Association of Kenya (AAK) with the partnership of the industry group CropLife—Kenya (Africa Middle East region) has established a PIC at the Kenyatta National Hospital in Nairobi. In 1996, CropLife Kenya commenced with the training of doctors for pesticide poisoning related issues but then expanded the information service to the general public. The PIC was launched in the same hospital in 2008. Nowadays, the focus on pesticides is lost and the center handles poisoning in general. The PIC is run by several stakeholders: CropLife Kenya, the hosting hospital and the Pharmaceutical Board of Kenya [33].

In Nairobi, the National Poison Information and Management Centre, at Gertrude's Children's Hospital carries out surveillance of toxic substances, with a focus on phytosanitary substances. The Center has an emergency phone line available to the public for free 24 h a day and seven days a week. Until 2010, the Center received on average 10 to 15 calls per day. The Center is equipped with the WHO/INTOX data management system for data collection and handling. 

In South Africa, TV activities are somewhat scattered. There are three PICs in the country: at the Red Cross War Memorial Children’s Hospital in Cape Town; the Tygerberg Hospital in Cape Town; and the University of Free State in Bloemfontein. All were started independently in the 1980s and each does things a little differently.

The Red Cross Children Hospital PIC has approximately 20 years of systematically collected data on all children admitted to this hospital for poisoning. The hospital also has data on telephone calls received on the emergency line from 2011. These data are used for publications in both local and international medical journals, and are made available to the public via the media, teaching campaigns and NGOs involved in preventive efforts. Where changes are detected in the pattern of poisoning or new agents emerge, the hospital alerts the relevant health authorities. Poison information activities started in 1971, using a card index and textbooks, and the PIC was established in 1984. A poison information database was designed as a result of a research project, and is now one of four poison databases in English worldwide and the only one in local language. The poison information database is called Afritox. Internet access is also required for the initial download of the database and intermittent for subsequent updates. Afritox includes relevant information on substances and treatment guidelines from international resources. There are only twoemergency phone lines available 24 h (sevendays/week) to the general public (both children and adults) and medical professions who both use this database as source of information. To date, it has grown from a system holding data on 200 to over 40,000 named toxics. The system is continuously updated to offer a better and more accessible poison advice service to medical professionals and the general public. 

An upgraded and Internet-based system will be accessible both on- and off-line by medical professionals throughout the entire southern African region. The web access to the database would facilitate medical doctors in their assistance to poisoned patients, provide the general public with information on non-toxic substances encountered on a daily basis, and relieve the load on the provincial health service and the poisons emergency line. It will ensure an improved and more accessible poison advice service, as well as a wide distribution of information. To date Afritox [34] has grown from a system holding data on 200 to over 40 000 substances, including commercial products, fauna and flora, and medicines It provides information on the ingredients, dosage, toxicity, symptoms and treatments, and much of the information is not available in standard medical textbooks.. Currently, the Red Cross War Memorial Children Hospital is the only institution that gathers and collates poisons information: its maintenance requires special skills, human resource, and equipment. 

In 2015, the emergency telephone services at Red Cross Children’s Hospital PIC and the Tygerberg PIC Centre were combined to provide one national number (+27-861-555-777). The service is available 24 h (7 days/week) to the general public and medical professionals who are advised according to the AfriTox database and other resources.

The Tygerberg PIC is situated in the Faculty of Health Sciences Stellenbosch University, in close proximity to the Tygerberg Academic Hospital, in the northern suburbs of Cape Town. The center provides a nationwide 24/7 telephone consultation service, in partnership with the Red Cross Children’s Hospital PIC as described above. Although the service primarily covers the Western Cape Province and neighboring regions, demands to meet national requirements have been increasing steadily over the past years. A similar cross-border tendency is evident, and service interaction with other countries on the African continent continues to escalate. Some typical enquiries are described below.

All data collected at the center was recorded into the WHO INTOX Data Management System, until the end of 2014. Since the introduction of the combined telephone service, details of calls are logged real-time into the custom-designed TeleLog database, which is linked to AfriTox. Data from the TeleLog are immediately available for TV activities, which is another first for Africa. Cases that need to be referred are followed up by the hospital consultant who makes the necessary telephone calls. Personnel of the center then link the follow-up calls to the original case on the database. This system enables the center to produce meaningful statistics for research purposes as well as a quarterly report for the hospital and the Department of Environmental Health.

The consultation service is geared to provide: (i) information on drugs, their indications and optimal applications in rational regimens for treating diseases; (ii) interpretation of the drug’s active ingredient levels in body fluids and their optimization in the course of therapeutic drug monitoring; (iii) general information on poisons, and potentially poisonous substances; (iv) general information on biological toxins, with special reference to poisonous and venomous creatures, poisonous plants and micro-organisms inhabiting the land, waterways and seas of the African continent; (v) interpretation of drugs’ active ingredients and xenobiotic levels in body fluids and tissues at autopsy (Forensic Toxicology); (vi) prioritization and procedures in cases of mass exposure to toxics and potential toxins, after transit and industrial accidents; (vii) level of alcohol, drugs and substances of abuse in body fluids of individuals to which situational statutory limits apply (e.g., drivers, operators of heavy machinery etc.), prohibited substances in sport; and (viii) expert advice in courts of law in the fields of pharmacology and toxicology, serving the needs of both the prosecution and the defense as dictated by circumstances.

The center provides a two-week training program for pharmacist interns, which in their turn are in charge of answering telephone calls. Trainees are coached on the correct procedures for answering and documenting calls, how to find the relevant data using our databases and reference books, and on the writing of reports. The center also provides a two-month Toxicology and Poison Information training course to Emergency Medicine and Clinical Pharmacology medical registrars.

In Zimbabwe, Tagwireri et al. [6] described the general pattern of poisoning. In a total of 2764 hospital admissions due to poisoning, accidental poisoning and deliberate self-poisoning accounted for 48.9% (1352 cases) and 41.3% (1142 cases), respectively. Within accidental poisoning cases, the highest number of cases (45.9%) occurred in children below the age of five years, with half of these due to chemicals, mainly paraffin. In the self-poisoning group, however, more than 60% of all cases occurred in the 16–25-year age group. Pesticides (31.4%) and pharmaceuticals (30.4%) were the most common groups of toxic agents responsible for hospital admissions. Unknown toxins, natural toxics and pesticides showed the highest mortality rates (15.4%, 8.3% and 6.7%, respectively). 

The Drug and Toxicology Information Service (DaTIS) is a unit within the School of Pharmacy at the University of Zimbabwe, College of Health Sciences, which is also supported by the Ministry of Health and Child Welfare as a mission set up in 1979. The unit is recognized by the WHO as a PIC and is listed as such on the WHO website. DaTIS has traditionally provided consultation to mostly health care professionals and, to a smaller extent, the general public on drug information and toxicological/poisoning related issues. Its poisons information service is provided nationally through a free 24-h telephone, seven days a week. During working hours, callers are given an immediate response whenever possible or by phone call returned as soon as possible. The unit also offers consultancy services on therapeutic drug monitoring/clinical pharmacokinetics and clinical analytical toxicology (measurement of medication levels in blood) through its therapeutic drug monitoring (TDM) unit based at Parirenyatwa Hospital. The main focus is on drugs with a narrow therapeutic range, i.e., drugs that can be easily overdosed. Its mission is amongst others to facilitate and promote safe use of chemicals and effective clinical management of poisoning through training of pre-registration pharmacists, teaching of pharmacists and other students on drug and poisons related issues, national and regional training of health personnel in pharmacotherapeutics and toxicology related issues, experimental toxicology research and consultancy in applied toxicology including TV, toxicoepidemiology, clinical, environmental and experimental toxicology [34].

### 2.4. Poison Control Centers: The Cases of Algeria, Morocco and Senegal 

The Algerian anti-poison center (CAP) has been operational since January 1991. The CAP was formally created with the national hospital reform of June 1998 [35]. The center is a public administration with financial autonomy. The CAP provides expertise on toxicology issues for the whole population within the country and work closely with emergency hospital units. The CAP is located at the toxicology laboratory of “Bab *el Oued”* University Teaching Hospital, and at the reanimation service of the “*Constantine*” University Teaching Hospital. Both of the units of the CAP are structured around four departments: medical toxicology, food toxicology, occupational toxicology, and ecotoxicology and environment.

Poisoning circumstances often identified are both accidental (carelessness, environmental pollution, and occupational exposure) and willful (suicide and criminal). Products most often incriminated for toxic risks are: drugs, household and industrial chemicals, pesticides, venom, cosmetics, plant products, contaminated food and gas. New poisoning cases are therefore declared and registered in specific forms designed by the center; they are thereafter kept at the documentation unit to constitute the database [36]. 

**Figure 1:**
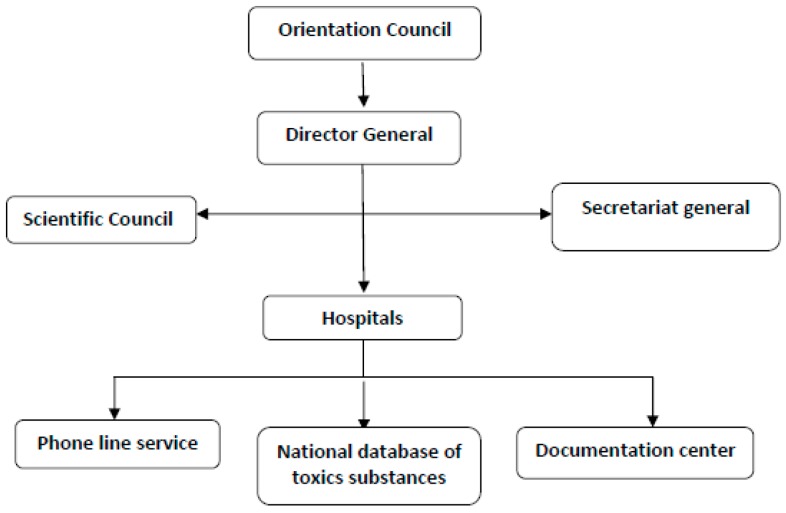
Organization chart of the Algeria national PCC.

Both the “Bab el Oued” and “Constantine” units work through: (i) a free phone line service; (ii) a national database of toxic substances; and (iii) the documentation center (Figure 1). The organization of the Algerian CAP allows proper interactions with populations to receive inquiries or provide advice; besides, the database and the documentation center help in collecting cases over time for analysis, as detailed in the following (Figure 1). 

The emergency free call number is available 24 h a day and seven days a week, for both inquiries on toxic chemicals and products, and declaration of poisoning cases. This number is at the disposition of health professionals and any other professional working with toxic products, decision makers, and the general public. Medical doctors are in charge of providing reliable response to callers. In case of declaration of poisoning, a form is available to appropriately describe clinical signs and help the responder establish a diagnosis and provide treatment. After each call is received, the medical doctor should produce an information sheet and computerize it; it is noted that received phone calls are recorded for eventual successive deeper analysis. The PCC does not provide medical treatment but contacts the nearest hospital emergency unit, or arrange home visits in minor cases. Apart from the free call service, declaration can also be done by fax or forms. The Ministerial ordinance N° 179/CAB of 17 November 1990 lists the diseases for which declaration is obligatory. Diseases listed are mostly infectious diseases; collective food toxi-infections (TIAC) are included. Once medical doctors diagnose a disease found on the list (suspected or confirmed), a declaration is made immediately. Those failing doing so are at risk of administrative and penal punishments. Responsible persons in charge of public or private laboratories should declare all confirmed diseases on the list. Notification of suspected or confirmed cases are made on specific declaration forms already printed and available in specialized hospitals, university teaching hospitals, and the competent office of the regional health sectors; some diseases have specific notification procedures. Offices of the health sector are in charge of the collection of declarations from laboratories and hospitals within their competencies and transmit them to the National Institute of Public Health in charge of the treatment, analysis and diffusion of national epidemiology statistics. The report established by the Institute is transmitted on a monthly basis to the Direction of prevention, Ministry of Health. Services of epidemiology and preventive medicine of the health sector ensure the implementation of prevention measures to fight diseases in the given sector. 

Primary data collected are exploited and if additional data are needed, epidemiology investigations are conducted, as well as exploitation of other existing international and national databases of toxic substances. The documentation unit is in charge of the elaboration of toxicology documents, and also constitutes a factual and bibliographic database that is updated on a regularly basis. This database contains relevant toxicology documentation and specialized publications [36]. A systematic report is published periodically. Information and knowledge gained are used to address crucial issues and launch alert actions to prevent future cases and protect populations. Reports are sent to the Ministry of Health, and recommendations are made on the ongoing epidemiological profile of intoxications as well as on major preventive and management actions taken, e.g., informing authorities and the general population, use restrictions, and security measures [37].

In 1989, the CAPM (Morocco poison control and pharmacovigilance center) already existed and was reorganized. Standard notifications forms were already used and annual report published. This is how the development of a real TV system was born [38]. With the Ministerial circular, cases received since 1980 were computerized for data exploitation. Information was used to establish a picture of the epidemiological profiles of intoxications in Morocco; the creation in 1990 of a free call number, available 24 h a day, seven days a week, facilitated the improvement of data collection directly from the population, the University teaching hospital, and private medical services. This facilitated a faster treatment of the declaration and notification process and deeper involvement of all stakeholders of the surveillance system. In 1992, the mission of the PCC was defined in the Ministerial circular N° 2DR/10: the CAPM is the national institution in charge of the management of poisoning issues at the individual and collective levels to decrease the morbidity and mortality due to intoxications. At the same time, it helps to rationalize health budget expenses.

**Figure 2:**
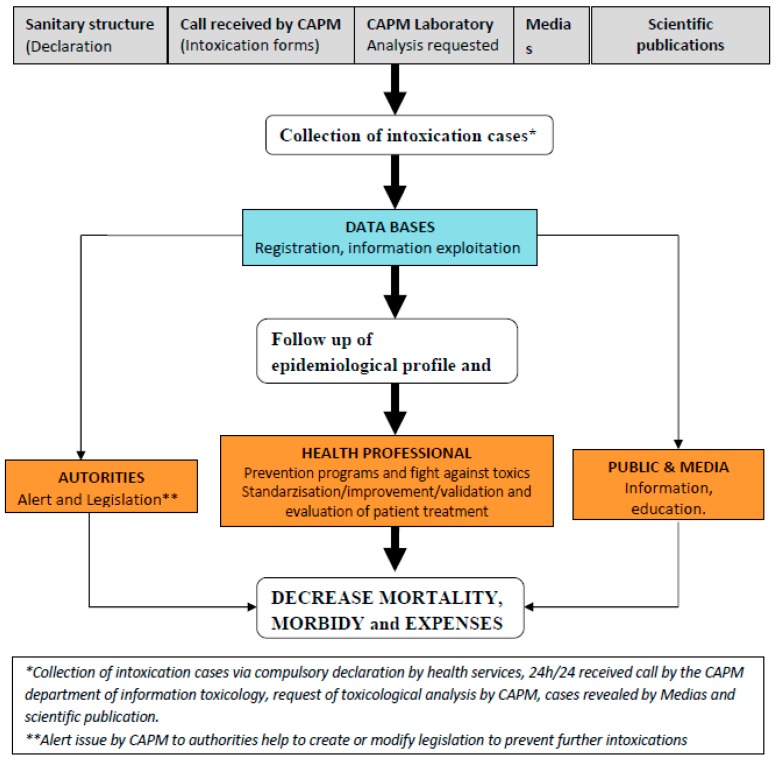
Work flow diagram of the TV system in Morocco.

The establishment of the first national epidemiological profile put into evidence the importance of scorpion bites and envenomation as the leading cause of intoxication in Morocco. For this reason, a more specific surveillance plan was put in place in 2001 to address snake bites issued by introducing a register for scorpion bites and envenomation cases. Interestingly, the TV system in Morocco has been greatly influenced by the national pharmacovigilance system; indeed, they function in tandem (Figure 2).

**Figure 3:**
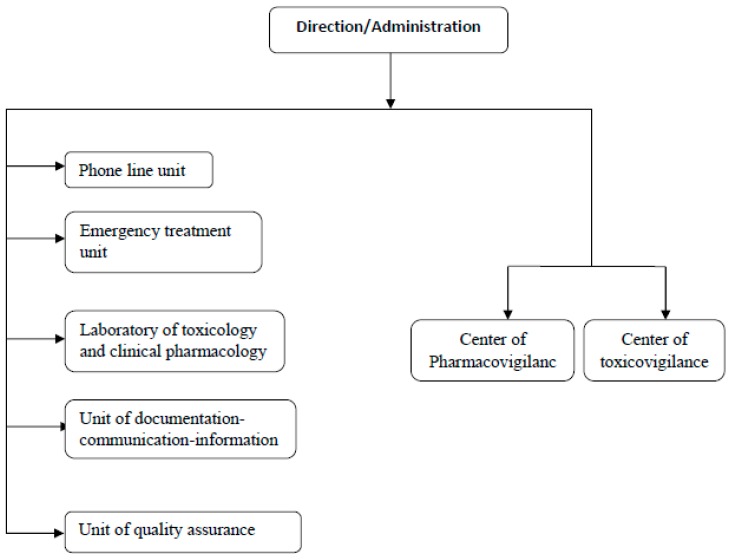
Organigram of the Toxicovigilance department at Morocco Poison Control and Pharmacovigilance Center.

The toxicovigilance activities pivot on information collection and database development and maintenance, as follows:

(**A**) The information collection service collects any information related to acute or chronic intoxication cases, potential toxic effects from natural, synthetic products or pollution through four information supports:
  **(1)**  A standard declaration form designed and used by medical services at regional and divisional levels to notify all intoxication cases except scorpion bites and envenomation (PES). The sub-titles of the form are: identification of the declaration source (region, division, and health service), characteristics of the intoxicate person (age, sex, profession, origin, and address), characteristics of the toxics (name, presentation, and dose), characteristics of the intoxications (date, type, single or collective, route of exposure, circumstances, place, clinical signs, para clinical tests, treatment, hospitalization and evolution). For each toxic agent, toxicological profiles are prepared and documented by the toxicology information unit to assist personnel to quickly respond to inquiries. All inquiries and request are analyzed to understand the ongoing trends.
  **(2)**  A system of specific information put in place in 2001 and possessing three information supports: register, monthly transcript, and hospitalization forms. This system is specifically used for scorpion bites and envenomation.
  **(3)**  Files for collective foods toxi-infections (TIAC) that should be automatically declared at the direction of epidemiology and fight against diseases, with the following parameters: (i) identification of the episode (region, dates, first cases, food consumption, declaration at the service of infrastructures and provincial ambulatory services, or SIAAP, and at the central services, duration of incubation, number of cases and clinical signs); (ii) suspected foods (nature, manipulation and mode of preparation, place of contamination and sampling, and sick person); (iii) fighting measures; and (iv) monitoring of the evolution of cases.
  **(4)**  Toxicology analysis request reaching the CAPM: these are written into a toxicology register and collected information help identify key variables to study intoxications. When needed, these data are completed with specific survey data, thesis, and other information released by scientific organizations or mass media. Data are reported on the standard form for declaration, after verification on the authenticity, elimination of doubling data and research of lack of data.
(**B**) The constitution of a national database for intoxications consists in the registration, exploitation and archives of poison information. In particular, declarations received are classified by region of origin and in chronological order; empty boxes are completed whenever possible and collective intoxications are identified. Remarks on the presentation, content of the forms, and therapeutic attitudes are noted. When necessary, a feed-back is given to the source of declaration. A bibliographic research is done for comparison with diagnostic etiology. Intoxication cases are therefore classified based on gravity of clinical symptoms (Poisoning Severity Score, PSS). Sensitization actions are regularly organized, through email address, fax, and phone conversation to improve the quality and reinforce notification services as well as recruitment of new sources of declarations. Data are sent to the statistic services for coding (internal thesaurus coding established by the center). Each variable receives a code and is typed using a typing mask. Archives are the final step in the data base constitution process; encoded data are kept order of arrival and by trimester.
  Databases from different Department of the center (Toxicovigilance Department, Information Technology Department and the laboratory) are compiled and statistically analyzed, thus supporting the update of the epidemiological profile of intoxications. Data analysis and exploitation start with a descriptive analysis of all parameters. Depending on the results, some data are analyzed with more details and alert actions are launched towards competent authorities (Ministry of Health, Ministry of Environment, the media, and other relevant agencies and institutions).
  Specific TV systems have been created; each system is under the responsibility of a group of persons in charge of follow-up, analysis and development of activities. Therefore, the general CAPM database has been subdivided in many specific databases depending on the nature of the toxic (e.g., plants, drugs, and cosmetics), the intoxicated person (e.g., children), and circumstances of intoxication (e.g., suicide with toxic products). This sub-division of databases helps in the detail analysis and identifies appropriate prevention actions to be taken.
  (Figure 3) underlined the general organization and activities at the Morocco TV department. Information and knowledge obtained from data analysis are used to produce a three-month report to be submitted during the quarterly meeting of the center. An annual report is also published in its official publication *La revue Toxicologie du Maroc*. When necessary, depending on information to be sent out and the targeted audience, alert activities are directed to health professionals concerning a toxic product or risk situation of exposure and diffusion. CAPM elaborates materials (e.g., leaflet, newsletter, CD-ROM, and poster), as well as training of health personnel on declaration and management of intoxication cases, and standardization in handling intoxication cases. Public health decision makers are reached through the publication of (annual) reports to help in formulating or modifying legislation on toxic compounds (e.g., the banning of the sale of paraphenylen diamine and mercury thermometers) or risk practices (e.g., the free sale of toxic plants/remedies by traditional doctors). The general public and the media are reached through information, education and communication campaigns at regional and national levels.

**Figure 4:**
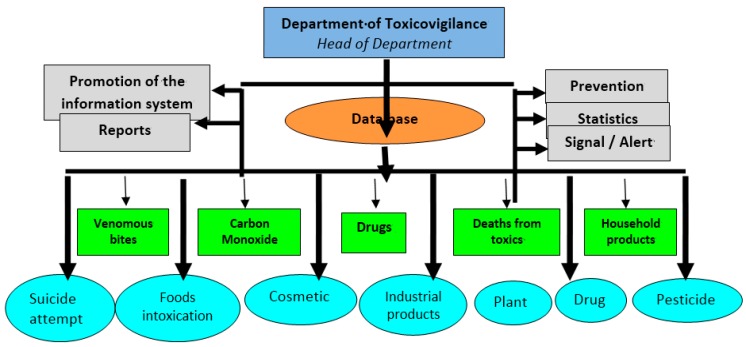
Organigram of the Senegal Poison Control Center.

In Senegal, the Ministerial decree N^o^ 611 MSP-DES of 29 January 2008 created and organized the national PCC. The center is established within “Fann” University Teaching Hospital of Dakar. The mission assigned to the center is the prevention and management of all sorts of intoxications (pesticides, household cleaning products, industrial products, plant products and other relevant xenobiotics). The center is organized in different services: (i) a phone line unit; (ii) an emergency treatment unit; (iii) a laboratory of toxicology and clinical pharmacology; and (iv) a TV center (Figure 4). The TV objective is to assist the government in its effort to reduce morbidity and mortality caused by toxic chemicals, elaborate the epidemiological profile of recorded intoxication cases through collection and centralization of declarations nationwide, and elaborate a prevention strategy and program to fight toxicant exposures.

The phone line is operational 24/7 to assist health professionals, regulators and consumers by providing appropriate response and guidance. The service is led by a medical doctor, specialized in emergency medicine. Phone calls can be done in official languages (French and Wolof) as well as other local vernacular languages (Serere, Toucouleur, Dioala, Manding, Soninke, Pular, etc.). Cases handled by the emergency treatment unit and the laboratory of toxicology and clinical pharmacology are collected and reported to the TV center for centralization. The TV center exploits different data to elaborate poisons epidemiological profiles. A report is periodically published and information is provided under request by the unit of documentation-communication-information. 

## 3. Discussion

In general, the TV system suffers from undernotification. Some health administrations in the private sector and Ministerial department need more sensitization campaigns on the collection and notification procedures (Municipal office of hygiene, military hospitals, etc.). In addition, intoxication cases that arrived in the public sector are not always declared. Even when considering the more mature TV system (in Morocco for example), the declaration mechanism is not regular and systematic enough despite the existence of Ministerial circulars. Moreover, the declaration system remains difficult to manage due to the hand filling of forms, registration, sending and late reception of declarations by courier. 

Action to improve/regularize the declaration mechanism could be: (i) automation by computerizing the information system; (ii) change the standard form with a section common to all toxics and a specific section depending on the toxics; and (iii) creation of regional TV centers to guarantee a better feed-back from regions and facilitate sensitization specific to the need of each region. Moreover, many poisoning cases cannot be confirmed by laboratory analysis, and call for an update of the clinical (emergency) toxicology facilities (from equipment and methods to international standards) both in central and peripheral regions.

Apart from Morocco, TV systems in Africa are generally solely based on toxicology data information, survey or surveillance results in a given period of time and a particular location for certain toxic agents. Without national coordination, the risk of activities overlapping by more than one institution is high (e.g., the case of collective food toxi-infections in Morocco is handled at the same time by CAPM, the Direction of epidemiology and fight against diseases, and the National Institute of Hygiene) and may generate confusion in declaration and duplication of cases. Furthermore, a national death register due to intoxications should be developed.

TV systems often derive from research programs and are useful as resource for research activity. Currently, they are used to improve toxicological knowledge through publications, collaborative projects and national and international scientific cooperation.

Some African TV systems, such as that operating in Morocco, represent a model for epidemiological surveillance. In a context where the identification of venomous species (snake, scorpion, spider, etc.) is still in an embryonic stage along with relevant national cartography of venomous animals and development of appropriate antidotes, the Morocco national strategy to fight against PES is a successful strategic model in Africa.

Furthermore, information sharing between all existing toxico-vigilance is a key point in building a regional TV system. The World Health Organization, Africa region organized in 2010 in Ghana a sub-regional workshop to discuss poisons center management and management of poisoning. Representatives from poisons centers in Ghana, Zimbabwe, Ghana, South Africa, as well as others representatives of countries without and established system. Activities to implement a Network of African Poison Centers and Applied Toxicologist (NAPCAT) were drafted. The network was to be register first in Ghana and South Africa as a not-for-profit association.

The SAICIM (Strategic Approach to International Chemicals Management) is a voluntary international agreement providing a policy framework to achieve the Johannesburg plan of Implementation (World Summit on Sustainable Development) in regards to sound chemicals management. In this project, establishment of toxico-surveillance systems is highly recommended. 

## 4. Conclusion

Toxicovigilance today is more reactive than preventive in Africa, but some results constitute some promising seminal milestones. The paper highlights the efforts made by the African continent so far to raise awareness and preparedness to chemical poisoning. 

Few countries are known to have a formal TV system (Morocco, Algeria, Republic of South Africa, Benin, Ghana, and Senegal), whereas others are on the way towards TV system through established PIC (e.g., Kenya and Zimbabwe). Countries without organized activity in the field (e.g., Cameroon) have, however, some mechanisms and practices in place for the surveillance of specific chemicals of concern, and a response plan in case of an outbreak situation. 

In general, basic structures include a phone line service (available 24 h a day, seven days a week), and hospital facilities. The existing surveillance models currently operational for some infectious diseases, zoonoses and chronic diseases constitute a good seed that could be exploited and extended to major toxicants and emerging risks. Risk analysis can guide the update of the list of substances and diseases under permanent vigilance, and for which notification to health authorities is obligatory. The overall scene of TV is rapidly changing in Africa, and calls for more, coordinated and harmonized efforts: the development of legal framework, the reinforcement of the capacity of analytical laboratories and other infrastructures, as well as trained manpower remains among major challenges. The harmonization with TV strategies adopted worldwide will also support Africa in protecting the health of its consumers and citizens through global health governance in the era of global trade, when formal and informal trade of external products will make the consumer exposed to new risks. Pesticides, including those dumped from economically advanced countries, emerge as the hazard of common interest in African TV systems, and may represent a prototypic toxicant towards the establishment of a TV system inclusive of a wider spectrum of toxicological hazards.

## Figures and Tables

**Table 1:** Examples of some poisoning cases reported in Africa.

Country	Poisoning Situation Products	Products and Chemicals	Reference
Algeria	Eating in events hall Eating in public restaurants Eating in University restaurant Herbal medicine	Meats and meats products, couscous, sweets	Albawaba news, 2005 [10]; Sebai and Boudali, 2007 [11]
Benin	Use of pesticide for plant treatment in cotton production	Callisulfan (endosulfan 350 g)	International POPs elimination Network, 2009
Cameroon	Gas eruption in the lac Nyos	Carbon dioxide	Tuttle et al., 1987 [12]
	Pesticides poisoning	Unknown	Sonchieu and Ngassoum, 2007 [13]
	University games restaurants	Unknown	Fred Vubem, 2007 [14]
	Eating street vended	popcorn	BBC, 2001 [15]
	Petroleum Bleach Drugs Rats poison	Unknown	Bulu, 1989 [16]
	Generating set	Carbon dioxide emission	Fomo, 2012 [17]
	Herbal products from traditional healer	Unknown	Fomo, 2012 [17]
Ghana	Pesticides poisoning	Pesticide which seeped into food stocks	NPAS, 2012 [18]
Kenya	Collective poisoning by porridge one day after funeral rites of a child (cassava and sorghum flour)	Hydrocyanic acid in cassava flour or oganophosphate contamination of the water used in preparing the porridge	Muruka et al., 2011 [19]
Morocco	Acute pesticide intoxication	Organophosphorus pyrethrinoides and carbamates	Idrissi et al., 2010 [20]
	Cosmetics poisoning (skin and hair products)	Para-phenylenediamine (PPD)	Semlali Hassani et al., 2011 [21]
Senegal	Lead poisoning	Batteries	Tall et al., 2010 [2]
	Scombroid fish poisoning occurred in the French Armed Forces in Dakar	Fish	Demoncheaux et al., 2012 [22]
South Africa	Paraffin (kerosene), drugs, household cleaning products, pesticides, cosmetics, household products, traditional medicine and environmental toxics	/	Kate Balm, 2012 [23], Muller et al., 1993 [24]
Zimbabwe	Accidental poisoning (children < 5) with paraffindeliberate self-poisoning	Pesticides and pharmaceuticals	Tagwireri et al. (2002) [6], Drug and Toxicology Information Services, (2012) [25]
